# Roles for endothelial cell and macrophage *Gch1* and tetrahydrobiopterin in atherosclerosis progression

**DOI:** 10.1093/cvr/cvy078

**Published:** 2018-03-27

**Authors:** Gillian Douglas, Ashley B Hale, Jyoti Patel, Surawee Chuaiphichai, Ayman Al Haj Zen, Victoria S Rashbrook, Lucy Trelfa, Mark J Crabtree, Eileen McNeill, Keith M Channon

**Affiliations:** 1 Division of Cardiovascular Medicine, BHF Centre of Research Excellence, Radcliffe Department of Medicine, John Radcliffe Hospital, University of Oxford, Oxford OX3 9DU, UK; 2 Wellcome Trust Centre for Human Genetics, University of Oxford, Roosevelt Drive, Oxford OX3 7BN, UK

**Keywords:** GCH, Tetrahydrobiopterin, Atherosclerosis, Macrophages, Endothelial cells

## Abstract

**Aims:**

GTP cyclohydrolase I catalyses the first and rate-limiting reaction in the synthesis of tetrahydrobiopterin (BH4), an essential cofactor for nitric oxide synthases (NOS). Both eNOS and iNOS have been implicated in the progression of atherosclerosis, with opposing effects in eNOS and iNOS knockout mice. However, the pathophysiologic requirement for BH4 in regulating both eNOS and iNOS function, and the effects of loss of BH4 on the progression of atherosclerosis remains unknown.

**Methods and results:**

Hyperlipidemic mice deficient in *Gch1* in endothelial cells and leucocytes were generated by crossing *Gch1*^fl/fl^Tie2cre mice with ApoE^–/–^ mice. Deficiency of *Gch1* and BH4 in endothelial cells and myeloid cells was associated with mildly increased blood pressure. High fat feeding for 6 weeks in *Gch1*^fl/fl^Tie2CreApoE^–/–^ mice resulted in significantly decreased circulating BH4 levels, increased atherosclerosis burden and increased plaque macrophage content. *Gch1*^fl/fl^Tie2CreApoE^–/–^ mice showed hallmarks of endothelial cell dysfunction, with increased aortic VCAM-1 expression and decreased endothelial cell dependent vasodilation. Furthermore, loss of BH4 from pro-inflammatory macrophages resulted in increased foam cell formation and altered cellular redox signalling, with decreased expression of antioxidant genes and increased reactive oxygen species. Bone marrow chimeras revealed that loss of *Gch1* in both endothelial cells and leucocytes is required to accelerate atherosclerosis.

**Conclusion:**

Both endothelial cell and macrophage BH4 play important roles in the regulation of NOS function and cellular redox signalling in atherosclerosis.

## 1. Introduction

GTP cyclohydrolase I (GTPCH) catalyses the first and rate-limiting reaction in the synthesis of tetrahydrobiopterin (BH4), an essential cofactor for the aromatic amino acid hydroxylases, alkylglycerol mono-oxygenase and the three nitric oxide synthases (NOS1–3).^1^ BH4 regulates NOS function, by coupling the haem-oxygen intermediate to l-arginine oxidation, thus controlling the generation of either nitric oxide (NO) or, in conditions of low BH4, superoxide, due to NOS uncoupling. In vascular disease states, BH4 bioavailability is decreased due to reduced biosynthesis,^2^ oxidative loss of BH4, forming dihydrobiopterin (BH2), and attenuated recycling of BH4 from BH2 by dihydrofolate reductase.^3^^,^^4^ In vascular disease states such as atherosclerosis, augmentation of endothelial cell BH4 levels, either by genetic over-expression of GTPCH or pharmacologic supplementation, can restore endothelial NOS function and reduce atherosclerotic plaque progression.^5^^,^^6^ However, the pathophysiologic requirement for endothelial cell BH4 biosynthesis in atherosclerosis, as distinct from the effects of BH4 supplementation, remain unknown.

A further important consideration in the role of NOS regulation by BH4 in atherosclerosis is the effect of BH4 on inducible NOS (iNOS) function. In atherosclerotic lesions iNOS co-localises with areas of lipid oxidation and protein nitration.^7^ iNOS expression exacerbates atherosclerotic plaque progression, with decreased atherosclerosis found in hyperlipidemic iNOS^–/–^ mice.^8–11^ This pro-inflammatory environment is predominantly driven by monocyte/macrophage infiltration, demonstrated in bone marrow chimera studies in iNOS^–/–^ mice in which leucocyte iNOS was shown to be pro-atherogenic.^12^ Inflammatory stimulation typically induces both iNOS expression, and increased BH4 biosynthesis.^13^^,^^14^ Patients with established atherosclerotic disease have reduced vascular BH4 levels, associated with impaired endothelial function, whereas plasma biopterins are increased, associated with biomarkers of inflammation.^15^^,^^16^ Thus, the requirement for BH4 in iNOS function in leucocytes is a novel and important consideration in the pathogenesis of atherosclerosis. However, previous studies in iNOS knockout mice only address the overall requirement for iNOS, and cannot address the importance of BH4 in iNOS function and the consequences of NOS uncoupling and redox dysregulation. Indeed, the opposing actions of endothelial cell eNOS vs. leucocyte iNOS in atherosclerosis, and the effects of BH4 in eNOS and iNOS function, have important implications for the potential of BH4 as a therapeutic target in vascular disease states.

We have recently generated a novel mouse model with conditional deletion of *Gch1*, leading to cell-specific loss of BH4 synthesis in endothelial cells and leucocytes. We sought to understand the requirement for BH4 in regulating both eNOS and iNOS, in endothelial and inflammatory cells, and the effects of loss of BH4 on the progression of atherosclerosis.

## 2. Methods

### 2.1 Animals

Endothelial and myeloid cell *Gch1* knockout mice on a hyperlipidemic (ApoE knock out; ApoE^–/–^) background were generated by crossing *Gch1*^fl/fl^Tie2cre mice^13^^,^^17^ with ApoE^–/–^ mice (Jackson Laboratories, Bar Harbor, USA). Matched litters of *Gch1*^fl/fl^Tie2CreApoE^–/–^ and *Gch1*^fl/fl^ApoE^–/–^ were generated by breeding male *Gch1*^fl/fl^Tie2CreApoE^–/–^ with female *Gch1*^fl/fl^ApoE^–/–^ females. Animals were housed in individually ventilated cages with 12-hour light/dark cycle and controlled temperature (20–22°C). Mice were fed standard chow (B&K Ltd, UK) up to 16 weeks of age or up to 10 weeks of age when they were switched to high fat diet (HFD; SDS 829108 Western RD diet, UK) for 6 weeks. Water and food were available *ab libitum* at all times.

Chimeric mice were generated in a manner similar to that described previously.^18^ Briefly, donor *Gch1*^fl/fl^ApoE^–/–^ and *Gch1*^fl/fl^Tie2CreApoE^–/–^ mice were killed and a single-cell suspension of bone marrow prepared. Six-week-old *Gch1*^fl/fl^ApoE^–/–^ and *Gch1*^fl/fl^Tie2CreApoE^–/–^ mice received a lethal dose of whole body irradiation (2 × 5 Gy) followed by an intravenous injection of 1 × 10^7^ bone-marrow cells in 0.2 mL phosphate-buffered saline from either Tie2cre-positive or negative donor mice. Four weeks (10 weeks of age) after bone marrow transplant chimerization was confirmed by PCR of tail blood and mice were switched to an HFD for 6 weeks. DNA was extracted from bone marrow at the time of harvest and the absence or presence of *Gch1* was assessed using PCR to confirm bone marrow reconstitution.

Genotyping of experimental mice was performed by standard PCR techniques. All mice were culled by exsanguination under terminal anaesthetic (isoflurane >3% in 95% O_2_ 5% CO_2_). All animal procedures were approved and carried out in accordance with the University of Oxford ethical committee and the UK Home Office Animals (Scientific Procedures) Act 1986. All procedures conformed with the Directive 2010/63/EU of the European Parliament.

### 2.2 Tissue collection

Tissue for histological analysis was collected from mice perfused with phosphate-buffered saline (PBS) followed by 4% paraformaldehyde, tissue for biochemical analysis was collected from mice perfused with PBS only and was snap frozen in liquid nitrogen and stored at –80°C until analysis. Primary endothelial cells were isolated from lungs by immunoselection with CD31 antibody (BD Biosciences, Wokingham, UK) coated magnetic beads as described previously.^19^ Bone-marrow–derived macrophages (BMDMs) were obtained as follows. Bone marrow was obtained by flushing the femur and tibia of adult mice with PBS. A single cell suspension was prepared by passing the bone marrow through a 70 mm cell strainer. Cells were cultured in 10 cm non-tissue culture treated dishes for 7 days in DMEM: F12 (Invitrogen, Loughborough, UK) supplemented with 100 U/mL penicillin and 100 ng/mL streptomycin (Sigma, Gillingham, UK), 10% (v/v) foetal bovine serum (PAA Laboratories, Loughborough, UK), 5 mM l-glutamine (Sigma), and 10–15% (v/v) L929 conditioned medium. The differentiation of the cells was confirmed using flow cytometry using a CyAn ADP (Beckton Coulter, High Wycombe, UK) for data acquisition and Flow Jo (TreeStar Inc., Wokingham, UK) for analysis. Macrophages were defined as being CD11b (PerCP conjugated) and F4:80 (APC conjugated, both Biolegend, London, UK) positive cells, as judged against isotype controls conjugated with the same fluorochromes (Biolegend). Following differentiation, cells were harvested and plated into 6- or 96-well plates containing serum-free media [Optimem supplemented with 100 U/mL penicillin and 100 ng/mL streptomycin and 0.2% (w/v) low-endotoxin bovine serum albumin (Sigma)]. Cells were stimulated with 10 ng/mL IFNγ (Peprotech EC) and 100 ng/mL LPS (Sigma) with or without acetylated LDL (20 μg/mL; Invitrogen) for 16 h; parallel wells were left unstimulated. After 16 h cell pellets and cell culture supernatants were collected, or the cells subjected to biochemical analysis.

Nuclear fractions were extracted from a total of 6 × 10^6^ macrophage using a nuclear fraction isolation kit (Cayman Chemicals, Ann Arbor, USA). Protein concentration in nuclear fractions was assessed using a modified Bradford assay. Nrf2 transcription activity of nuclear fractions (6 μg total nuclear protein) was quantified by assessing transcription factor binding activity (Cayman Chemicals).^20^

Total RNA was extracted using the Ambion Pure Link kit. Reverse transcription was carried out using QuantiTect reverse transcription kit (Qiagen, Hilden, Germany, UK) on 1 μg total cell RNA. Quantitative real-time RT–PCR was performed with an iCycler IQ real-time detection system (BioRad Laboratories, Hercules, USA) using primers and probes from the TaqMan Gene Expression Assay system (Life Technologies, Loughborough, UK). Gene expression data were normalized to GAPDH with the exception of BMDM when β-actin was used.

### 2.3 Western blotting

Western blotting was carried out on aorta, primary endothelial cells, BMDM homogenates (15 µg protein), liver (5 µg protein), and macrophage nuclear fraction (5 µg nuclear protein) using standard techniques and iNOS (BD Pharmigen, Wokingham, UK, 610329), anti-GTPCH (custom made, a gift from Dr S. Gross), GAPDH (Millpore, Watford, UK MAB374), TBP (Abcam, Cambridge, UK; ab818), and Nrf2 (Abcam, Cambridge, UK; ab137550) antibodies.

### 2.4 Isometric tension vasomotor studies

Vascular rings were isolated from thoracic aorta of female chow and HFD mice and mounted on a wire myograph (MultiMyogrph 610M, Danish Myo Technology, Aarhus, Denmark) containing Krebs-Henseleit buffer. Vessel viability was tested using 60 mM KCl. Concentration–response contraction curves were established to phenylephrine and acetylcholine in the absence and presence of 10 µM, L-NAME or 10 µM sepiapterin.

### 2.5 Determination of BH4 levels

BH4 levels in tissue, cells, and plasma were determined by high-performance liquid chromatography (HPLC) followed by electrochemical and fluorescent detection, as previously described.^4^

### 2.6 Quantification of superoxide production

Superoxide production from primary endothelial cells and BMDM from 16-week-old chow fed mice was measured by quantifying the accumulation of 2-hydroxyethidium by HPLC as previously described.^4^

### 2.7 Blood pressure and pulse measurements in conscious mice

Heart rate and systolic blood pressure were measured using an automated computerized tail-cuff system in 16-week-old chow and HFD fed conscious mice, as described previously (Visitech BP2000, Visitech Systems, Inc., Apex, USA).^21^

### 2.8 Lipid and lipoprotein analysis

Biochemical analyses of plasma lipids were performed on heparinized blood plasma using enzymatic assays. BMDM were differentiated into macrophages as above. Cells were cultured in Optimem containing 0.2% fatty acid free serum and labelled with [^3 ^H] cholesterol (2 µCi/mL) overnight. Cells were washed and equilibrated overnight and reverse cholesterol transport to pooled ApoE^–/–^ plasma (2.5%) assessed after 6 h by scintillation counting of media and cellular [H^3^] cholesterol. For lipid uptake assays, cells BMDM were either unstimulated or stimulated with LPS/IFNγ as above. After stimulation overnight, cells were treated with 20 µg/mL of Dil AcLDL (Invitrogen, Loughborough, UK) for 8 h. Fixed cells were imaged using the Operetta high content imaging system (Perkin Elmer, Seer Green, UK). The fluorescent intensity resulting from cellular uptake of DiI AcLDL was quantified using Harmony image analysis software.

### 2.9 Histology and immunohistochemistry of atherosclerotic aortic root sections

Lesion size was assessed in paraffin-embedded aortic root sections stained with Masson’s-Goldner (Merck, Darmstadt, Germany). Average lesion size was calculated from six sections taken at 45 µm intervals starting from the section showing all three aortic cusps. Aortic lesion macrophage content was analysed using anti-Gal-3 (BD Pharmingen, Wokingham, UK) immunostaining. Plaque collagen content was assessed using Sirius red staining. Mast cells within the plaque and perivascular area of the aortic root were assessed by Toludine Blue staining. Lesion area, Gal-3, and collagen positive areas were quantified from digitized microscopic images using Image-Pro Plus.

### 2.10 Nitrite determination and analysis of NO synthesis

Nitrite accumulation was measured in samples of cell culture medium using the Griess assay with colorimetric detection in 96-well plates, as described.^22^ NO synthesis activity was assessed by conversion of [14C]l-arginine to citrulline, in the presence and absence of *N*-methyl-Larginine, as described previously.^23^

### 2.11 Quantification of plasma leucocytes and peritoneal macrophage recruitment

Cell populations in whole blood (EDTA) from high fat fed mice were stained with monoclonal antibodies directed against CD45^+^, CD11b^+^, Ly6C^+^, Ly6G^–^ (monocytes) and CD45^+^, CD11b^+^, Ly6C^+^, Ly6G^+^ (neutrophils). For *in vivo* peritoneal recruitment experiments mice were injected ip with 4% thioglycolate, 4 days later mice were killed and the peritoneal cavity was lavaged with 5 mL of PBS containing 2 mM EDTA. Peritoneal exudate cells were stained with antibodies against CD45 (all leucocytes), CD11b and F4:80 (macrophages). All flow cytometry was performed using a DAKO CyAn cytometer and Summit software (Beckton Coulter, High Wycombe, UK). Data was analysed using Flow Jo software (TreeStar Inc, Wokingham, UK). Where cells were enumerated an absolute count protocol was used, where cells were quantified by ratio to a known number of fluorescent beads which were spiked into the sample prior to analysis.

### 2.12 Statistical analysis

Data are presented as mean ± SEM. Normality was tested using D’Agostino and Pearson omnibus normality test. Groups were compared using the Mann–Whitney *U* test for non-parametric data or an un-paired Student’s *t*-test for parametric data. When comparing multiple groups data were analysed by analysis of variance (ANOVA) with Newman–Keuls post-test for parametric data or Kruskal–Wallis test with Dunns post-test for non-parametric data. When more than two independent variables were present a two-way ANOVA with Tukey’s multiple comparisons test was used. When within subject repeated measurements were present a repeated measures (RM) ANOVA was used. A value of *P* < 0.05 was considered statistically significant.

## 3. Results

### 3.1 Leucocyte and endothelial cell *Gch1* deficiency leads to decreased circulating BH4 and increased arterial blood pressure after high fat feeding

**Figure 1:**
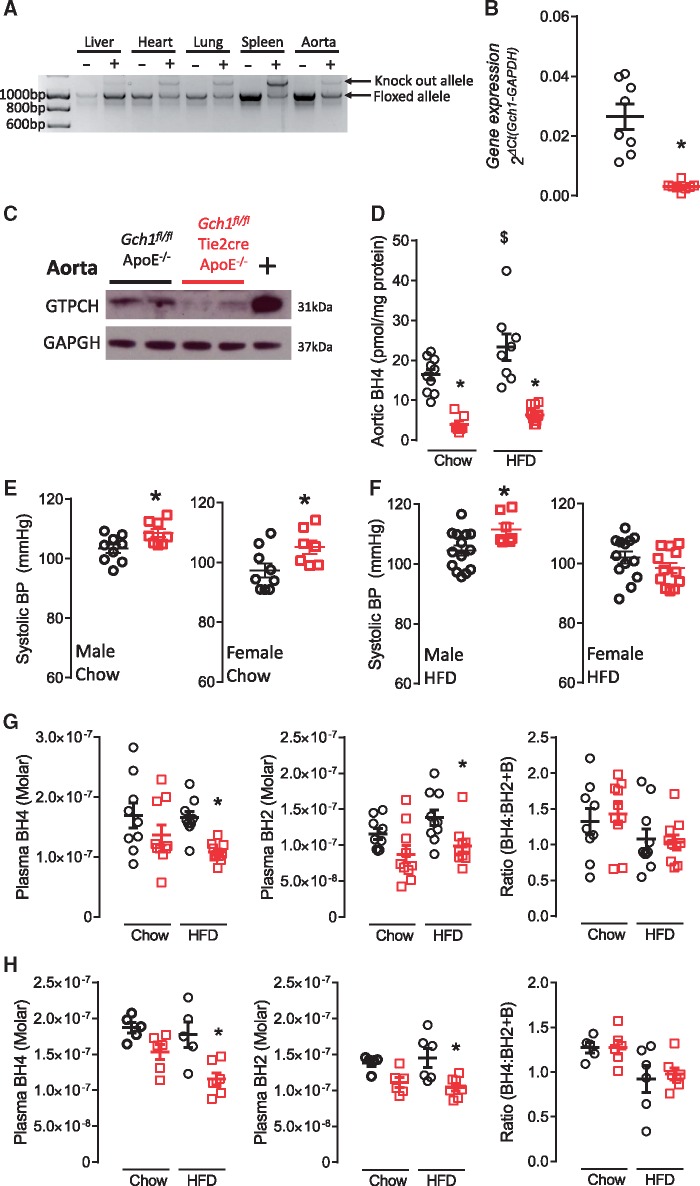
Characterization of the *Gch1*^fl/fl^Tie2CreApoE^–/–^ mouse. (*A*) PCR genomic DNA in tissues from *Gch1*^fl/fl^ApoE^–/–^ and *Gch1*^fl/fl^Tie2CreApoE^–/–^ mice. (*B*) Real time qRT–PCR showing a significant decrease in expression in aortas from 16-week-old non-HFD female *Gch1*^fl/fl^Tie2CreApoE^–/–^ mice compared with aortas from *Gch1*^fl/fl^ApoE^–/–^ littermates (un-paired *T*-test, *n* = 8 per group). (*C*) Representative immunoblot showing decreased expression of GTPCH in aortas from *Gch1*^fl/fl^Tie2CreApoE^–/–^ mice. LPS/IFNγ stimulated BMDMs are used as a positive control. (*D*) BH4 in aortas of chow or HDF mice showing a significant decrease in BH4 levels in *Gch1*^fl/fl^Tie2CreApoE^–/–^ and a significant increase in BH4 levels in *Gch1*^fl/fl^ApoE^–/–^ after HFD (two-way ANOVA, *n* = 7–12 per group). (*E*) Systolic blood pressure was increase in chow fed *Gch1*^fl/fl^Tie2CreApoE^–/–^ mice compared with *Gch1*^fl/fl^ApoE^–/–^ (un-paired *T*-test; *n* = 7–9 per group). (*F*) Systolic blood pressure was increase in male HFD *Gch1*^fl/fl^Tie2CreApoE^–/–^ male mice but not females (un-paired *T*-test, *n* = 7–14 per group). BH4, BH2, and the ratio of BH4:BH2 and B in plasma from chow and HFD male (*G*) and female (*H*) mice. No difference in plasma levels between genotypes was observed in chow few mice, but a significant decrease in plasma BH4 and BH2 levels were observed in *Gch1*^fl/fl^Tie2CreApoE^–/–^ mice after HFD (two-way ANOVA; *n* = 5–10 per group). Data are expressed as the mean ± SEM. *P < 0.05 between genotypes of the same treatment. ^$^*P* < 0.05 between treatments of the same genotype. Black symbols = *Gch1*^fl/fl^ApoE^–/–^, red symbols = *Gch1*^fl/fl^Tie2CreApoE^–/–^.

Genomic PCR demonstrated efficient excision of the floxed *Gch1* allele in endothelial and myeloid cell-rich tissues such as aorta, lungs, heart and spleen from *Gch1*^fl/fl^Tie2CreApoE^–/–^ mice, compared with minimal excision in liver (*Figure 1A*). Endothelial and leucocyte specific *Gch1* deletion resulted in a significant reduction in aortic *Gch1* expression (>90%; *P* < 0.05; *Figure 1B*) and GTPCH protein (*Figure 1C*). Accordingly, BH4 levels were reduced by more than 80% in whole aortas (16.39 ± 1.38 vs. 3.96 ± 0.80 pmol/mg protein, *P* < 0.05, *Figure 1D*). Interestingly, a significant increase in aortic BH4 and BH2 content was observed in HFD *Gch1*^fl/fl^ApoE^–/–^ mice, whereas no difference in BH4 or BH2 content was observed in aortas from HFD vs. chow fed *Gch1*^fl/fl^Tie2CreApoE^–/–^ (*Figure 1D* and Supplementary material online, *Figure* S*1A*).

We next assessed how endothelial cell and leucocyte-specific *Gch1* deficiency altered blood pressure in hyperlipidemic ApoE^–/–^ mice. At 16 weeks of age systolic blood pressure was slightly higher in both male (103 ± 1.5 vs. 109 ± 1.3 mmHg) and female (97 ± 2 vs. 105 ± 2.3 mmHg) chow fed *Gch1*^fl/fl^Tie2CreApoE^–/–^ mice compared with their control littermates (*P* < 0.05, *Figure 1E*). This increase in blood pressure was preserved in HFD male mice, however, in HFD female mice the increase in blood pressure observed in chow fed *Gch1*^fl/fl^Tie2CreApoE^–/–^ was no longer apparent (*Figure 1F*). There was no difference in heart rate in either chow or HFD males or female mice (Supplementary material online, *Figure S2*). In mice fed a standard chow diet, circulating levels of BH4 and BH2 in male and female *Gch1*^fl/fl^Tie2CreApoE^–/–^ were not significantly different compared with their matched littermates. However, after 6 weeks on an HFD plasma levels of both BH4 and BH2 levels were significantly reduced in both male (*Figure 1G*) and female (*Figure 1H*) *Gch1*^fl/fl^Tie2CreApoE^–/–^ mice (*P* < 0.05). The similar reduction in BH4 and BH2 in *Gch1*^fl/fl^Tie2CreApoE^–/–^ mice, resulted in no change in the ratio of BH4 to BH2 and B. As the liver is hypothesized to be the major site of circulating BH4, we assessed the impact of loss of *Gch1* in endothelial cells and leucocytes on liver levels. The absence of *Gch1* did not impact on either GTPCH protein or BH4 levels in the livers of chow fed mice (Supplementary material online, *Figure* S*1B* and *C*). However, after 6 weeks of HFD a significant reduction in BH4 levels was observed in livers from *Gch1*^fl/fl^Tie2CreApoE^–/–^ mice (Supplementary material online, *Figure* S*1C*). In contrast, a significant increase in liver BH2 was observed after HFD in both groups, with a greater increase observed in livers from *Gch1*^fl/fl^ApoE^–/–^ mice (Supplementary material online, *Figure* S*1D*).

### 3.2 Deficiency in *Gch1* in leucocytes and endothelial cells results in increased atherosclerosis burden

We next aimed to assess how *Gch1* deletion and BH4 deficiency in endothelial cells and leucocytes altered the development of atherosclerosis. Male and female *Gch1*^fl/fl^Tie2CreApoE^–/–^ and *Gch1*^fl/fl^ApoE^–/–^ littermates were fed an HFD for 6 weeks from 10 to 16 weeks of age. There was no difference in body weight between the two groups (*Table 1*). Plasma lipid analysis revealed that loss of *Gch1* in endothelial cells and leucocytes did not alter circulating levels of either total cholesterol, LDL cholesterol, triglycerides, or HDL. Furthermore, there was no change in circulating leucocytes between groups, with no difference observed in numbers of monocytes (CD45^+^, CD11b^+^, Ly6C^+^, and Ly6C^–^) or neutrophils (CD45^+^, CD11b^+^, Ly6C^+^, and Ly6G^+^; *Table 1*).

**Table 1:** Plasma lipid levels and circulating leucocyte levels in the high fat fed *Gch1*^fl/fl^ApoE^–/–^ and *Gch1*^fl/fl^Ties2CreApoE^–/–^ mice

	Males		Females	
	*Gch1*^fl/fl^ApoE^–/–^	*Gch1*^fl/fl^Tie2Cre ApoE^–/–^	*Gch1*^fl/fl^ApoE^–/–^	*Gch1*^fl/fl^Tie2Cre ApoE^–/–^
Body weight (g)	36.7 ± 1.0	37.9 ± 1.3	25.6 ± 0.8	25.3 ± 1.0
Total cholesterol (mmol/L)	46.64 ± 2.09	43.76 ± 1.40	38.44 ± 1.79	33.13 ± 1.52
LDL cholesterol (mmol/L)	31.66 ± 1.20	30.83 ± 0.84	26.08 ± 1.66	24.37 ± 1.00
Triglycerides (mmol/L)	4.17 ± 0.28	3.65 ± 0.26	2.08 ± 0.29	2.67 ± 0.35
HDL cholesterol (mmol/L)	2.95 ± 0.11	2.88 ± 0.09	2.13 ± 0.07	2.24 ± 0.07
Monocytes (cells/mL blood)	2.558 × 10^5^ ± 1.073 × 10^5^	5.365 × 10^5^ ± 1.388 × 10^5^	1.958 × 10^5^ ± 2.067 × 10^4^	2.491 × 10^5^ ± 2.193 × 10^4^
Neutrophils (cells/mL blood)	2.787 × 10^5^ ± 5.161 × 10^4^	4.109 × 10^5^ ± 7.988 × 10^4^	2.010 × 10^5^ ± 7.995 × 10^3^	2.364 × 10^5^ ± 1.144 × 10^4^

Data are presented as the mean ± SEM, *n* = 5 for leucocytes analysis and 6–10 per group for lipid analysis.

**Figure 2:**
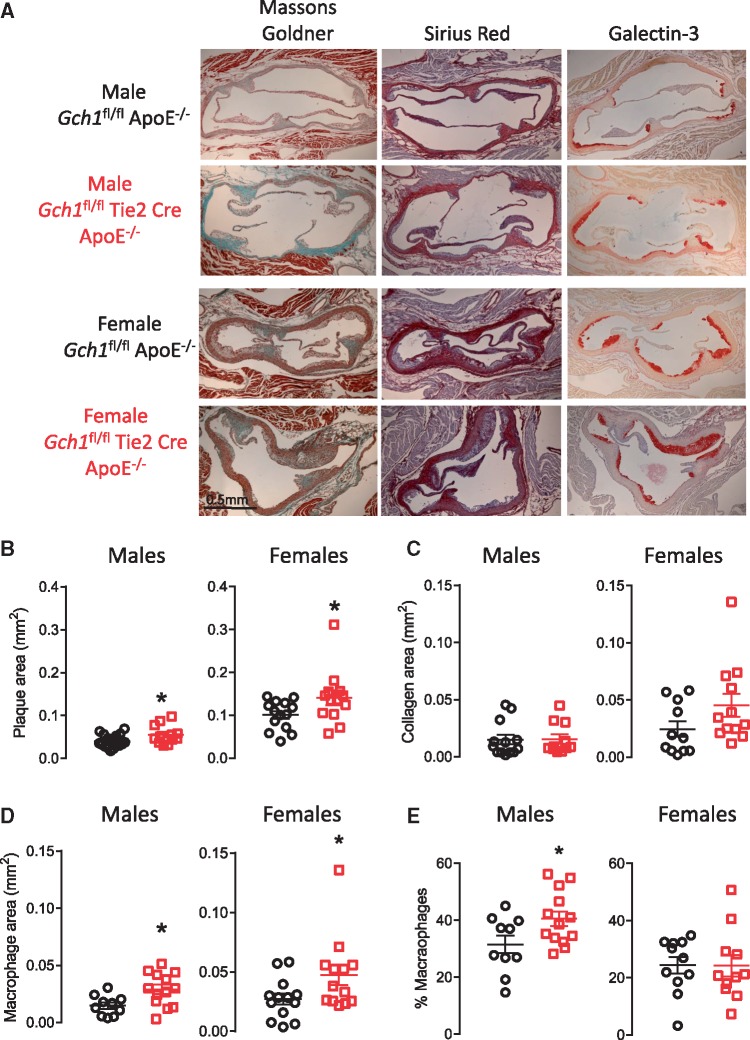
Loss of *Gch1* in endothelial cells and leucocytes causes increased atherosclerosis. Quantification of aortic root atherosclerosis in *Gch1*^fl/fl^ApoE^–/–^ and *Gch1*^fl/fl^Tie2CreApoE^–/–^ mice at 16 weeks after 6 weeks of HFD (10–16 weeks of age). (*A*) Representative picture of aortic roots stained with Masson’s Goldner for plaque quantification, Sirius red for collagen content and Galectin-3 for macrophage content (macrophages stained red). (*B*) A significant increase in plaque was observed in both male and female *Gch1*^fl/fl^Tie2CreApoE^–/–^ compared with *Gch1*^fl/fl^ApoE^–/–^ (*P* < 0.05) as assessed by Masson’s Goldner staining. (*C*) Sirius red staining was not different between genotypes (*P* > 0.05). (*D*) A significant increase in plaque macrophage content was observed in aortic roots from both male and female *Gch1*^fl/fl^Tie2CreApoE^–/–^ mice (*P* < 0.05). (*E*) A significant increase was observed in percentage macrophage content in plaques from male *Gch1*^fl/fl^Tie2CreApoE^–/–^ but not female mice (*P* < 0.05). Data are expressed as the mean ± SEM, un-paired *T*-test between different genotypes of the same sex; *n* = 12–18 per group *P < 0.05 between genotypes of the same treatments. Black symbols = *Gch1*^fl/fl^ApoE^–/–^, red symbols = *Gch1*^fl/fl^Tie2CreApoE^–/–^, black scale bar = 0.5 mm.

Analysis of Masson-Goldner stained aortic roots (average of six sections, taken 45 µm apart) demonstrated a significant increase in plaque burden in aortic roots from *Gch1*^fl/fl^Tie2CreApoE^–/–^ mice in both males (0.040 ± 0.003 vs. 0.055 ± 0.006 mm^2^, *P* < 0.05) and females (0.100 ± 0.009 vs. 0.141 ± 0.017 mm^2^, *P* < 0.05; *Figure 2A* and *B*). Although absolute plaque size was different in males vs. females, a similar percentage increase in plaques size was observed in both sexes (28% and 27%, respectively). Collagen content, assessed with Sirius red staining, was not significantly different in plaques from *Gch1*^fl/fl^Tie2CreApoE^–/–^ mice and their littermate controls (*Figure 2A* and *C*). Total mast cell content of the aortic root, assessed in Toluline Blue stained sections, was not significantly different between groups. In addition, there was no difference in the number of granulated or de-granulated mast cells in aortic roots between *Gch1*^fl/fl^Tie2CreApoE^–/–^ mice and their littermate controls (Supplementary material online, *Figure S3*).

Immunostaining for macrophages (Galectin-3; Gal-3) indicated that the increase in plaque size was accompanied by an increase in plaque macrophage content in *Gch1*^fl/fl^Tie2CreApoE^–/–^ mice in both males (0.014 ± 0.002 vs. 0.030 ± 0.004 mm^2^, *P* < 0.05) and females (0.023 ± 0.04 vs. 0.047 ± 0.008 mm^2^, *P* < 0.05; *Figure 2A* and *D*). In female mice, there was no difference in the percentage of macrophage content in the plaques, indicating that the increase in macrophage content was accompanied by a proportionate increase in plaque size. However, in male mice there was a significant increase in the percentage macrophage content of the plaques (31.51 ± 3.15 vs. 40.47 ± 2.61%, *P* < 0.05) indicating that plaques from male *Gch1*^fl/fl^Tie2CreApoE^–/–^ mice were more inflamed than plaques from *Gch1*^fl/fl^ApoE^–/–^ (*Figure 2E*).

### 3.3 Endothelial cell deficiency in BH4 leads to enhanced vasoconstriction, impaired vasodilation, and endothelial cell dysfunction

**Figure 3:**
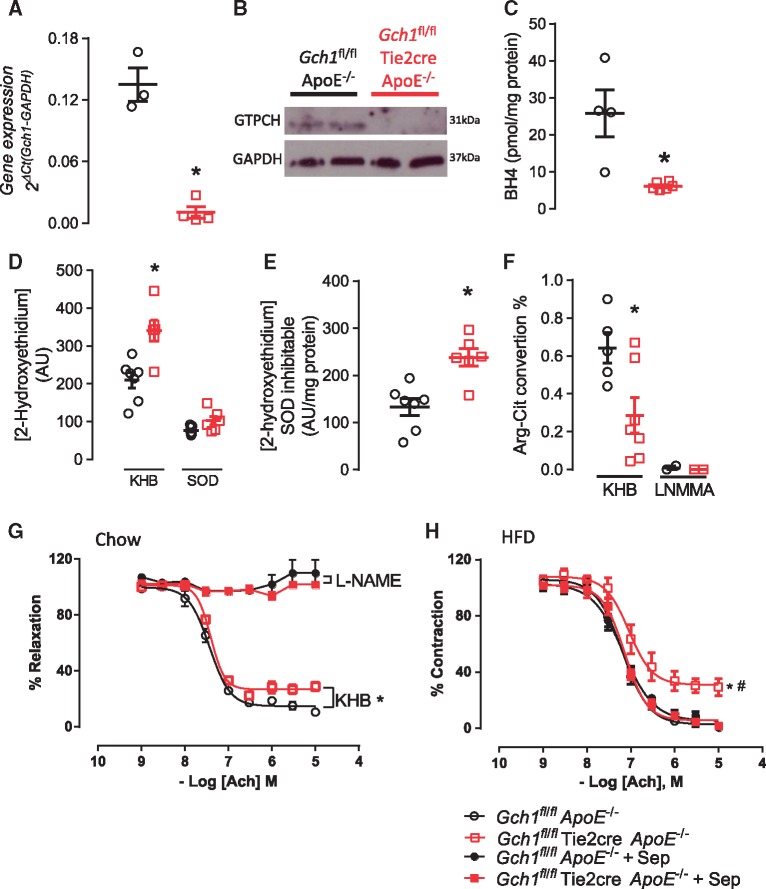
Characterization of endothelial cells from *Gch1*^fl/fl^ApoE^–/–^ and *Gch1*^fl/fl^Tie2CreApoE^–/–^mice. (*A*) Real time qRT–PCR showing a significant decrease in *Gch1* expression in primary endothelial cells from *Gch1*^fl/fl^Tie2CreApoE^–/–^ mice (Mann–Whitney *U* test; *n* = 3–4 per group). (*B*) Representative immunoblot of GTPCH levels in primary endothelial cells showing decreased expression of GTPCH in endothelial cells from *Gch1*^fl/fl^Tie2CreApoE^–/–^ mice. (*C*) BH4 levels in primary endothelial cells were significant reduced in endothelial cells from *Gch1*^fl/fl^Tie2CreApoE^–/–^ mice (Mann–Whitney *U* test, *n* = 4–6 per group). (*D*) Superoxide generation in primary lung endothelial cells from *Gch1*^fl/fl^ApoE^–/–^ and *Gch1*^fl/fl^Tie2CreApoE^–/–^ mice, endothelial cells from *Gch1*^fl/fl^Tie2CreApoE^–/–^ mice had a significant increase in 2-hydroxyethidium (two-way ANOVA; *n* = 5–7 per group). (*E*) SOD (100 U/mL) caused a significant decrease in 2-hydroxyethidium production in both groups with a greater inhibitable fraction observed in endothelial cells from *Gch1*^fl/fl^Tie2CreApoE^–/–^ (Mann–Whitney *U* test; *n* = 6–7 per group). (*F*) Quantification of arginine to citrulline conversion in primary endothelial cells from HFD mice. A significant decrease in conversion was observed in cells from *Gch1*^fl/fl^Tie2CreApoE^–/–^ (un-paired *T*-test, *n* = 6–8 in KHB, *n* = 2 representative aortas in the LNMMA group). (*G*) Endothelial dependent vasodilation to acetylcholine (Ach) was significantly reduced in aortas from chow fed female *Gch1*^fl/fl^Tie2CreApoE^–/–^ mice, incubation with L-NAME completely inhibited relaxation in both groups (RM ANOVA; *n* = 7 per group). (*H*) HFD caused a significant decrease in endothelial dependent vasodilation to Ach in aortas from female *Gch1*^fl/fl^Tie2CreApoE^–/–^ mice, *ex vivo* incubation with sepiapterin restored the dilator response to wild type levels (RM ANOVA, *n* = 8 per group). *P < 0.05 between genotypes of the same treatments. Black symbols = *Gch1*^fl/fl^ApoE^–/–^, red symbols = *Gch1*^fl/fl^Tie2CreApoE^–/–^, KHB = Krebs Henseleit buffer.

We next evaluated the requirement for BH4 in primary endothelial cells from *Gch1*^fl/fl^Tie2CreApoE^–/–^ mice. Both *Gch1* RNA expression and GTPCH protein levels were significantly reduced (*P* < 0.05; *Figure 3A and B*), leading to greatly reduced BH4 and BH2 levels in endothelial cells from *Gch1*^fl/fl^Tie2CreApoE^–/–^ compared with *Gch1*^fl/fl^ApoE^–/–^ (25.83 ± 6.33 vs. 6.13 ± 0.40, *Figure 3C* and Supplementary material online, *Figure* S*1E*).

To determine the effects of endothelial cell-specific BH4 deficiency on endothelial cell function, we first measured basal O_2_^–^ production in isolated primary endothelial cells. Generation of 2-hydroxyethidium from hydroxyethidine was significantly higher in primary endothelial cells from *Gch1*^fl/fl^Tie2CreApoE^–/–^ mice (209 ± 20 vs. 340 ± 28; *Figure 3D*). Using superoxide dismutase (SOD) to confirm the specificity of the 2-hydroxyethidium product for O_2_^–^; we also observed a greater SOD inhabitable fraction of 2-hydroxyethidium in endothelial cells from *Gch1*^fl/fl^Tie2CreApoE^–/–^ (*P* < 0.05; *Figure 3E*).

We next assessed NOS activity using radiolabelled l-arginine to l-citrulline conversion. Primary endothelial cells from HFD *Gch1*-deficient mice showed a significant decrease in l-arginine to l-citrulline conversion compared with endothelial cells from their control littermates. The specificity for NOS activity was confirmed using the NOS inhibitor LNNMA, in both groups (*P* < 0.05; *Figure 3F*).

We next investigated how endothelial cell BH4 deficiency in ApoE^–/–^ mice affected vascular function. Isometric tension studies in isolated aortas from chow fed *Gch1*^fl/fl^Tie2CreApoE^–/–^ mice demonstrated a significant increase in phenylephrine-induced vasoconstriction and a significant reduction in endothelial-dependent vasorelaxation to acetylcholine. The relaxation response to acetylcholine in both groups was completely abolished in the presence of L-NAME, confirming NOS as the major source of vasodilators (*Figure 3G* and Supplementary material online, *Figure* S*4A*). Endothelial cell dependent relaxation response to acetylcholine was significantly blunted in aortas from HFD *Gch1*^fl/fl^Tie2CreApoE^–/–^ mice, but this effect was reversed in the presence of sepiapterin (*Figure 3H*). The enhanced constrictor response observed in chow fed *Gch1*^fl/fl^Tie2CreApoE^–/–^ mice was not observed in HFD mice. There was no difference in constrictor response between the two genotypes after treatment with sepiapterin (Supplementary material online, *Figure S4B*).

**Figure 4:**
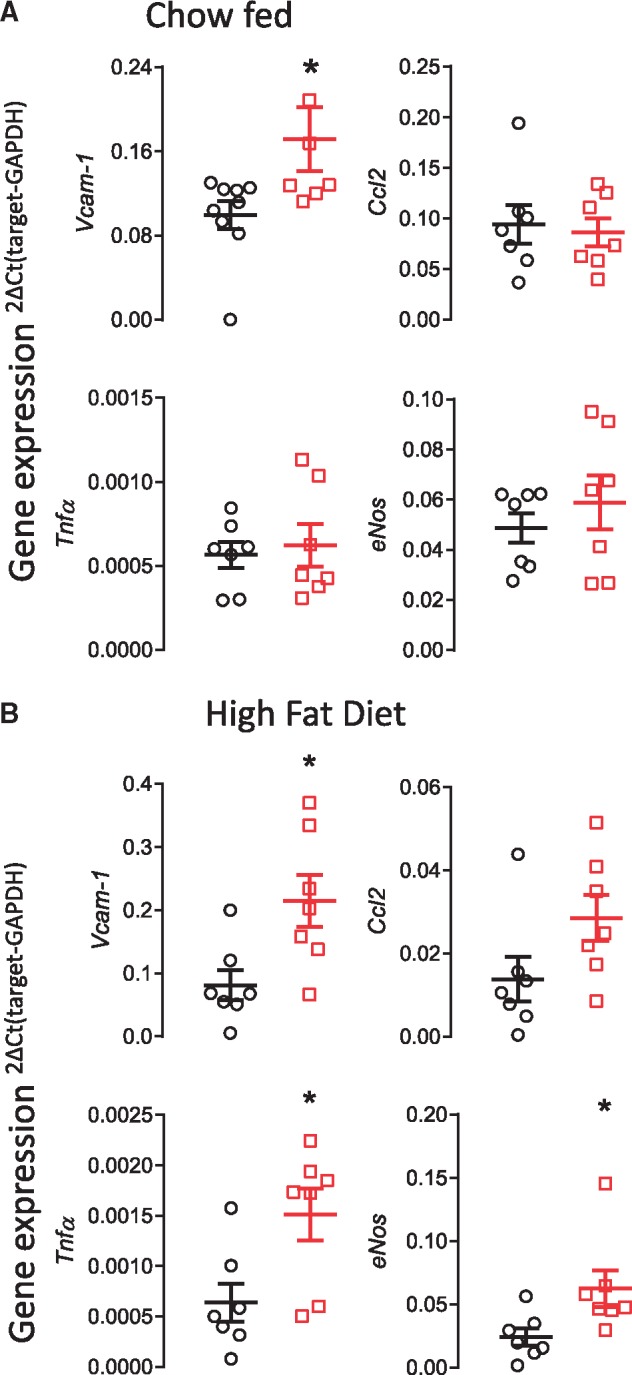
Loss of *Gch1* leads to increased aortic expression of atherosclerosis related genes. (*A*) Increase in VCAM-1expression in aortas from 16-week-old chow fed female *Gch1*^fl/fl^Tie2CreApoE^–/–^ mice compared with their *Gch1*^fl/fl^ApoE^–/–^ littermates. There was no difference between genotype in expression of either CCL2, eNOS or TNF-α. (*B*) A significant increase in VCAM-1, eNOS and TNF-α was observed in aortas from HFD *Gch1*^fl/fl^Tie2CreApoE^–/–^. No significant difference in CCL2 was observed (*P* = 0.053). Data are expressed as the mean ± SEM, Mann–Whitney *U* test, *n* = 6–9 per group, *P < 0.05 between genotypes of the same treatment. Black symbols = *Gch1*^fl/fl^ApoE^–/–^, red symbols = *Gch1*^fl/fl^Tie2CreApoE^–/–^.

We next assessed how deficiency in endothelial cell *Gch1* altered aortic expression of pro-atherogenic genes. Aortic *Vcam1* expression was significantly higher in chow fed *Gch1*^fl/fl^Tie2CreApoE^–/–^ mice (0.1 ± 0.01 vs. 0.17 ± 0.03; *P* < 0.05 *Figure 4A*). Deficiency in *Gch1* in chow fed mice did not lead to a compensatory change in eNOS levels, nor did it not alter expression of either CCL2 or TNFα (*Figure 4A*). *Vcam1* levels were also significantly elevated in aortas from HFD *Gch1*^fl/fl^Tie2CreApoE^–/–^ mice and, in contrast to aortas from chow fed mice, a significant increase in both TNF-α and eNOS was observed along with a non-significant trend towards an increase in CCL2 (*P* = 0.053, *Figure 4B*).

### 3.4 Macrophages from *Gch1*^fl/fl^Tie2CreApoE^–/–^ mice have altered iNOS function and increased ROS generation

**Figure 5:**
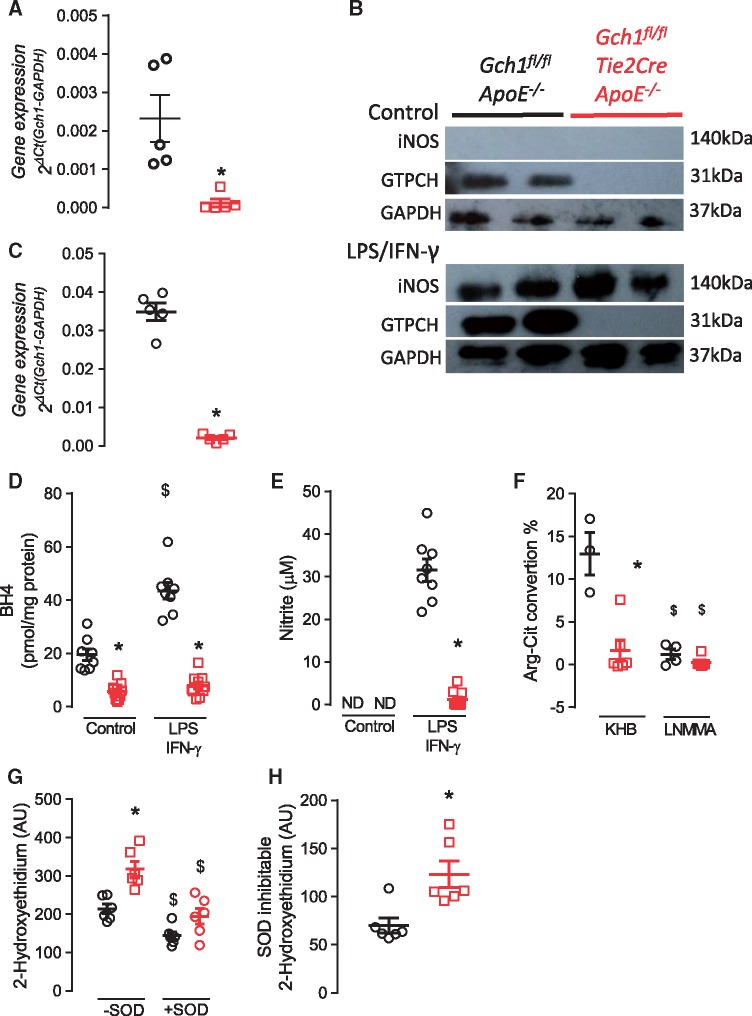
Characterization of BMDM from *Gch1*^fl/fl^ApoE^–/–^ and *Gch1*^fl/fl^Tie2CreApoE^–/–^ mice. (*A*) Real time qRT–PCR expression of *Gch1* in unstimulated macrophages showing a significant decrease in *Gch1* expression in *Gch1*^fl/fl^Tie2CreApoE^–/–^ macrophages (Mann–Whitney *U* test, *n* = 5 per group). (*B*) Representative immunoblot showing iNOS and GTPCH levels in stimulated (LPS/IFNγ) and unstimulated macrophages. (*C*) Real time qRT–PCR expression of *Gch1* in LPS/IFNγ stimulated macrophages showing a significant decrease in *Gch1* expression in *Gch1*^fl/fl^Tie2CreApoE^–/–^ macrophages (Mann–Whitney *U* test, *n* = 5 per group). (*D*) BH4 levels in unstimulated and LPS/IFNγ stimulated macrophages, a significant reduction in BH4 levels was observed in macrophages from *Gch1*^fl/fl^Tie2CreApoE^–/–^ mice (two-way ANOVA, *n* = 8 per group). (*E*) Nitrite accumulation in the cell culture media over 18 h of cell stimulation using the Greiss assay. Stimulation with LPS/IFNγ cause a significant increase in nitrite in macrophages from *Gch1*^fl/fl^ApoE^–/–^ mice however levels were only just detectable in macrophages from *Gch1*^fl/fl^Tie2CreApoE^–/–^ mice (two-way ANOVA, *n* = 8 per group). (*F*) Quantification of arginine to citrulline conversion in LPS/IFNγ stimulated macrophages. A significant decrease in conversion was observed in macrophages from *Gch1*^fl/fl^Tie2CreApoE^–/–^, LNNMA cause a significant decrease in arginine to citrulline conversion in both groups (two-way ANOVA, *n* = 3–6 per group). (*G*) Superoxide generation in LPS/IFNγ stimulated macrophages from *Gch1*^fl/fl^ApoE^–/–^ and *Gch1*^fl/fl^Tie2CreApoE^–/–^ mice, macrophages from *Gch1*^fl/fl^Tie2CreApoE^–/–^ mice had a significant increase in 2-hydroxyethidium production. SOD (100 U/mL) caused a significant decrease in 2-hydroxyethidium production in both groups (two-way ANOVA, *n* = 6–7 per group) with a significantly greater inhabitable fraction observed in macrophages from *Gch1*^fl/fl^Tie2CreApoE^–/–^ (*H*, Mann–Whitney *U* test, *n* = 6–7 per group). Data are expressed as the mean ± SEM, *P < 0.05 between genotypes of the same treatment, ^$^*P* > 0.05 between treatments of the same genotype. Black symbols = *Gch1*^fl/fl^ApoE^–/–^, red symbols = *Gch1*^fl/fl^Tie2CreApoE^–/–^.

We next sought to determine the requirement for *Gch1*, and BH4, in macrophages from ApoE^–/–^ mice. Deletion of *Gch1* resulted in a greater than 90% loss of *Gch1* mRNA and no detectable GTPCH protein in BMDMs (*Figure 5A* and *B*). Stimulation of BMDM for 24 h with LPS/IFNγ resulted in upregulation of *Gch1* in macrophages from control *Gch1*^fl/fl^ApoE^–/–^ mice, which was accompanied by a robust increase in iNOS protein (*Figure 5B*). However, despite iNOS being similarly upregulated in macrophages from both groups, *Gch1* mRNA remained significantly decreased in macrophages from *Gch1*^fl/fl^Tie2CreApoE^–/–^ mice (0.034 ± 0.0022 vs. 0.002 ± 0.0004, *P* < 0.05) with GTPCH protein levels undetectable by western blot (*Figure 5B* and *C*). Consequently, BH4 levels in both unstimulated and stimulated and BH2 levels in stimulated macrophages from *Gch1*^fl/fl^Tie2CreApoE^–/–^ mice were strikingly reduced (*P* < 0.05; *Figure 5D* and Supplementary material online, *Figure S1F*). We next assessed how this deficiency in *Gch1* and BH4 altered the ability of the macrophages to generate nitric oxide. Nitrite levels were undetectable in unstimulated macrophages from either genotype. However, upon stimulation with LPS/IFNγ, macrophages from control mice accumulated very high levels of nitrite in the medium, whereas BH4-deficient macrophages from *Gch1*^fl/fl^Tie2CreApoE^–/–^ mice generated minimal nitrite (31.8 ± 5.4 vs. 1.9 ± 00.9, *P* > 0.05; *Figure 5E*). A similar result was observed using radiolabelled l-arginine to l-citrulline conversion, in order to directly quantify NOS enzymatic activity. LPS/IFNγ-stimulated macrophages from *Gch1* deficient mice showed minimal l-arginine to l-citrulline conversion compared with macrophages from their control littermates (*Figure 5F*). The specificity for NOS activity was confirmed using the NOS inhibitor L-NMMA, in both groups (*P* < 0.05; *Figure 5F*).

To investigate how deletion of *Gch1*, loss of BH4 and altered iNOS activity affects macrophage ROS production, we next measured O_2_^–^ in LPS/IFNγ stimulated macrophages. 2-Hydroxyethidium production was significantly increased in *Gch1* deficient macrophages, indicating elevated O_2_^–^ production (214 ± 30.5 vs. 317 ± 19.8, *P* < 0.05; *Figure 5G*). In addition the SOD inhabitable fraction was significantly greater in macrophages from *Gch1*^fl/fl^Tie2CreApoE^–/–^ mice (70.0 ± 7.8 vs. 123.1 ± 13.8, *P* < 0.05; *Figure 5H*).

### 3.5 Loss of BH4 increases macrophage foam cell formation and modifies cellular redox signalling

**Figure 6:**
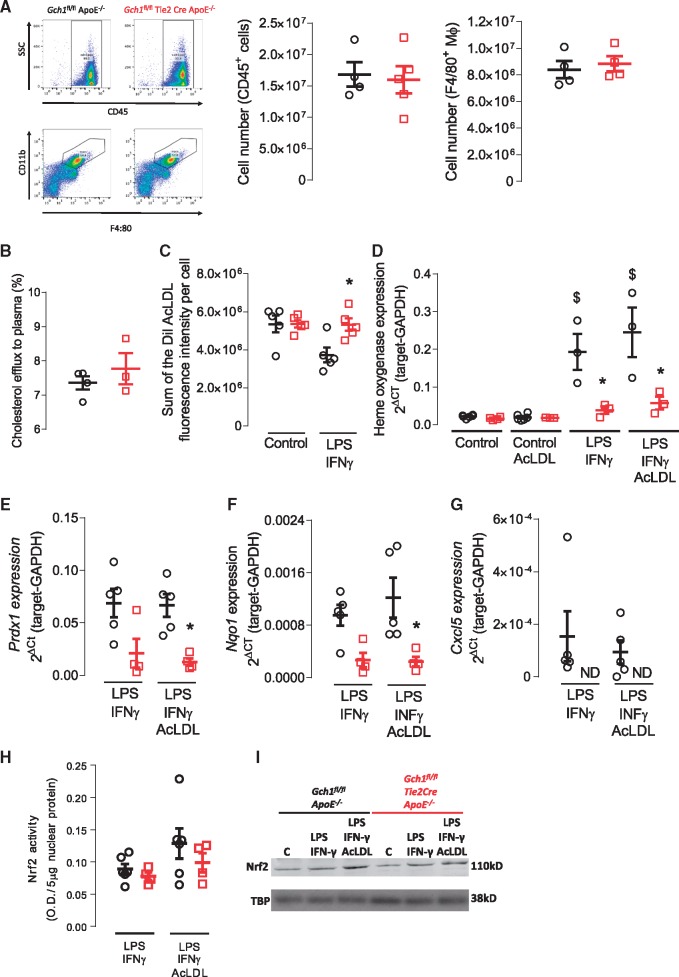
Loss of *Gch1* in BMDM leads to increased foam cell formation and anti-oxidant signalling. (*A*) Representative flow cytometry dot plots showing side scatter and cell surface expression of CD45 and CD11b and F4:80. Quantification of CD45^+^ cells and CD11b^+^ and F4:80^+^ macrophages in peritoneal lavage from thioglycolate treated mice showed no difference between the two genotypes (Mann–Whitney *U* test, *n* = 4–5). (*B*) Reverse cholesterol transport to pooled ApoE^–/–^ plasma in unstimulated BMDM from *Gch1*^fl/fl^ApoE^–/–^ and *Gch1*^fl/fl^Tie2CreApoE^–/–^ mice, no significant differences were observed between groups (Mann–Whitney *U* test, *n* = 3–4 per group). (*C*) Foam cell formation in unstimulated and LPS/IFNγ stimulated BMDM, no difference between groups was observed in unstimulated macrophages; however, a significant increase in foam cell formation was observed in LPS/IFNγ stimulated macrophages from *Gch1*^fl/fl^Tie2CreApoE^–/–^ mice (two-way ANOVA, *n* = 5 per group). (*D*) Real time qRT–PCR in unstimulated and LPS/IFNγ stimulated macrophages fed AcLDL (20 µg/mL). No difference in heme oxygenase expression was observed in unstimulated macrophages, stimulation with LPS/IFNγ resulted in a significant increase expression in macrophages from *Gch1*^fl/fl^ApoE^–/–^; however, this augmentation was significantly reduced in macrophages from *Gch1*^fl/fl^Tie2CreApoE^–/–^ mice (two-way ANOVA, *n* = 4–5 per group). Real time qRT–PCR to Prdx1 (*E*), Nqo1 (*F*), and Cxcl5 (*G*) in LPS/IFNγ stimulated macrophages with or without AcLDL (20 µg/mL). For all genes, a significant reduction in expression was observed in AcLDL treated macrophages from *Gch1*^fl/fl^Tie2CreApoE^–/–^ mice (two-way ANOVA, *n* = 4–5 per group). (*H*) Nrf2 nuclear transcription assay in LPS/IFNγ stimulated macrophages with or without AcLDL (20 µg/mL). No significant difference was observed in nuclear Nrf2 activity between groups (one-way ANOVA, *n* = 5 per group). (I) Representative western blot showing Nrf2 levels in unstimulated (*C*) and LPS/IFNγ ± AcLDL stimulated BMDMs. Data are expressed as the mean ± SEM, *P < 0.05 between genotypes of the same treatment, ^$^*P* > 0.05 between control and treatment of the same genotype. Black symbols = *Gch1*^fl/fl^ApoE^–/–^, red symbols = *Gch1*^fl/fl^Tie2CreApoE^–/–^.

We next sought to determine the effects of BH4-dependent iNOS function in macrophages, in order to explore the mechanisms underlying increased atherosclerotic plaque formation in *Gch1*^fl/fl^Tie2CreApoE^–/–^ mice. We first assessed *in vivo* chemotaxis in to the peritoneal cavity in response to an acute inflammatory response (4% thioglycollate). There was no difference in the number of CD45^+^ cells between *Gch1*^fl/fl^Tie2CreApoE^–/–^ and control littermates (*P* > 0.05; *Figure 6A*). We next characterized the recruited cells, using CD11b and F4:80 to identify macrophages. We found no significant difference in the number of peritoneal macrophages between the two groups (*P* > 0.05; *Figure 6A*), suggesting that loss of BH4, and NO production, in LPS/IFNγ stimulated macrophages does not significantly alter chemotactic responses *in vivo*.

To investigate how *Gch1* deficiency affects lipid handling, we assessed the capacity of macrophages from *Gch1*^fl/fl^Tie2CreApoE^–/–^ mice to form foam cells and efflux cholesterol. There was no difference in reverse cholesterol transport from macrophages to plasma between *Gch1*^fl/fl^Tie2CreApoE^–/–^ and *Gch1*^fl/fl^ApoE^–/–^ mice (*P* > 0.05, *Figure 6B*). We next used fluorescently labelled acetylated low-density lipoprotein (AcLDL) to assess foam cell formation. Foam cell formation in unstimulated macrophages was unchanged in macrophages from *Gch1*^fl/fl^Tie2CreApoE^–/–^ mice. However, in inflammatory LPS/IFNγ stimulated macrophages *Gch1* deficiency resulted in a significant increase in foam cell formation (*P* < 0.05; *Figure 6C*).

Dysregulation of the oxidative pathway have previously been shown to alter foam cell formation. We used qRT–PCR to quantify expression of anti-oxidant genes in unstimulated and LPS/IFNγ stimulated macrophages. We found that treatment with LPS/IFNγ caused a significant up regulation of heme oxygenase in both genotypes. However, upregulation of heme oxygenase was strikingly reduced in macrophages from *Gch1* deficient mice compared with control *Gch1*^fl/fl^ApoE^–/–^ mice (0.19 ± 0.048 vs. 0.039 ± 0.010, *P* < 0.05). Treatment of macrophages with AcLDL did not alter the expression of heme oxygenase (*Figure 6D*). Reduced expression of *Prdx1* and *Nqo1* was also observed in *Gch1* deficient macrophages (*Figure 6E* and *F*). In addition, we also observed a striking absence in the expression of the chemokine receptor *Cxcl5* in *Gch1*-deficient LPS/IFNγ stimulated macrophages treated with LPS/IFN-γ and AcLDL (*Figure 6G*). In order to assess whether the differences observed in anti-oxidant gene expression was due to alteration in the translocation of Nrf2 to the nucleus, we assessed Nrf2 transcription activity in nuclear fractions from LPS/INF-γ and LPS/IFN-γ and AcLDL treated macrophages. We observed no differences in Nrf2 reporter activity in nuclear fractions from either LPS/IFN-γ or LPS/IFN-γ and AcLDL treated macrophages between *Gch1* deficient and control macrophages (*Figure 6H*). Western blot analysis showed a similar finding to the activity assay with no difference between genotypes observed (*Figure 6I*).

### 3.6 Deficiency in BH4 in both endothelial cells and leucocytes is required to increase atherosclerosis burden

Given the changes observed in both endothelial cell and macrophage biology in the absence of *Gch1* we next sought to determine the contributions of these cell types to the observed increase in atherosclerosis in *Gch1*^fl/fl^Tie2CreApoE^–/–^ mice. Three groups of bone marrow chimeric animals were generated: (1) *Gch1*^fl/fl^Tie2CreApoE^–/–^ were given bone marrow from *Gch1*^fl/fl^ApoE^–/–^ to assess the impact of loss of *Gch1* from endothelial cells on atherosclerosis progression; (2) *Gch1*^fl/fl^ApoE^–/–^ received *Gch1*^fl/fl^Tie2CreApoE^–/–^ bone marrow to assess the role of leucocyte *Gch1*; or (3) *Gch1*^fl/fl^ApoE^–/–^ received *Gch1*^fl/fl^ApoE^–/–^ bone marrow, as a control group.

**Figure 7:**
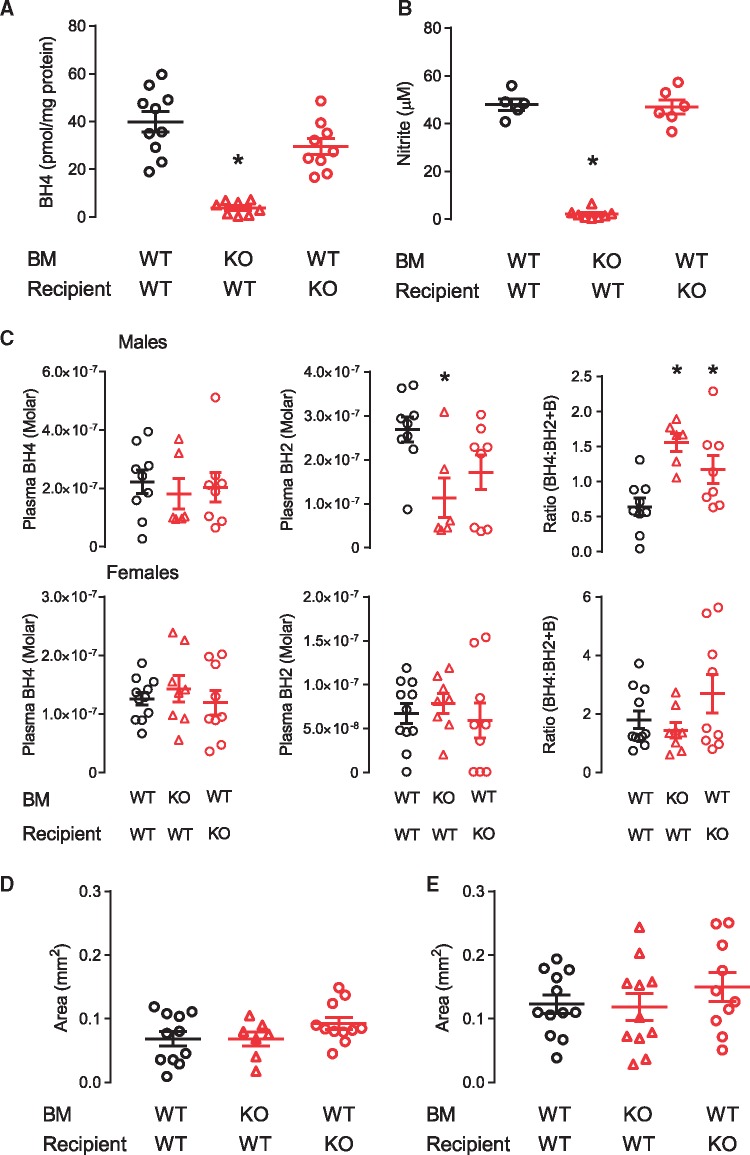
Loss of *Gch1* in both endothelial cells and leucocytes is required to increase atherosclerosis. (*A*) BH4 levels and (*B*) nitrite accumulation in LPS/IFNγ stimulated BMDM, a significant reduction in BH4 and nitrite levels was observed in macrophages from *Gch1*^fl/fl^ApoE^–/–^ recipient mice who received bone marrow from a *Gch1*^fl/fl^Tie2CreApoE^–/–^ donor (Kruskal–Wallis test, *n* = 5–10 per group). (*C*) BH4, BH2, and the ratio of BH4:BH2 and B in plasma from chimeric male (Kruskal–Wallis test) and female (one-way ANOVA) mice. No difference in plasma BH4 levels were observed between genotypes. In male mice a significant reduction in BH2 was observed in mice with a leucocyte deficiency in *Gch1*, with no difference observed between groups in female mice. In males a significant increase in the ratio of BH4:BH2 and B was observed in both endothelial cells and leucocytes *Gch1* deficient mice, no difference were observed between groups in female mice (*n* = 5–11 per group). Quantification of aortic root atherosclerosis at 16 weeks after 6 weeks of HFD in male (*D*, Kruskal–Wallis test) and female (*E*, one-way ANOVA) mice, no difference in plaque area was observed between groups in either males or females (*n* = 7–12 per group). *P < 0.05 compared with control. Black symbols = *Gch1*^fl/fl^ApoE^–/–^, red symbols = *Gch1*^fl/fl^Tie2CreApoE^–/–^.

BMDMs from chimeric mice were assessed for BH4 levels and nitrite production to confirm appropriate reconstitution with donor marrow. As expected, both BH4 and nitrite levels in BMDM followed the genotype of the bone marrow donor. *Gch1*^fl/fl^ApoE^–/–^ mice which received bone marrow from *Gch1*^fl/fl^Tie2CreApoE^–/–^ mice had a significant reduction in both BH4 and nitrite, whereas BH4 and nitrite levels in BMDM from *Gch1*^fl/fl^Tie2CreApoE^–/–^ which received *Gch1*^fl/fl^ApoE^–/–^ bone marrow where similar to the levels observed in the control mice (*Gch1*^fl/fl^ApoE^–/–^ which received *Gch1*^fl/fl^ApoE^–/–^ bone marrow, *Figure 7A* and *B*). We next quantified plasma biopterin levels in chimeric mice. In male mice, loss of *Gch1* from leucocytes caused a significant reduction in plasma BH2 levels but with preserved BH4 levels (*Figure 7C*). The preservation of BH4 levels and the reduction in both BH2 and B (data not shown) resulted in a significant increase in BH4:BH2 + B ratio in both endothelial cells and leucocyte knockout mice. In contrast, no difference in either BH4, BH2 or the ratio was observed between any of the groups of female mice (*Figure 7C*).

**Figure 8:**
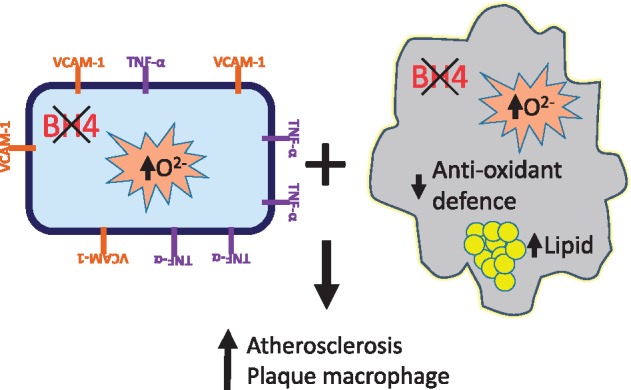
Loss of *Gch1* in both endothelial cells and monocyte/macrophages is required to increase atherosclerosis burden.

We next assessed the impact of loss of *Gch1* in either endothelial cells or leucocytes on atherosclerotic plaque in aortic root sections. Loss of *Gch1* from either endothelial cells or leucocytes alone did not alter atherosclerotic plaque burden in either male or female mice, indicating that deficiency of *Gch1* from both endothelial cells and leucocytes is required to increase atherosclerosis burden (*Figures 7D* and *E* and *8*).

## 4. Discussion

We have generated a novel mouse model of endothelial cell and leucocyte-targeted *Gch1* deletion, crossed with the hyperlipidemic ApoE^–/–^ mouse, to test the cellular requirement for BH4 in the pathology of atherosclerosis. This novel mouse model reveals several new and important findings. First, deficiency in endothelial cell and leucocyte BH4 results in an increase in atherosclerotic plaque formation. Second, *Gch1*^fl/fl^Tie2Cre ApoE^–/–^ mice display hallmarks of endothelial cell dysfunction characterized by loss of NO-mediated effects. Third, loss of BH4 in inflammatory macrophages alters lipid handling, leading to increased foam cell formation and alteration in anti-oxidant dependent gene expression. Finally, loss of *Gch1* in both endothelial cells and leucocytes was required to increase atherosclerotic burden. Our study highlights the consequences of loss of both endothelial cell and macrophage BH4 in the regulation of NOS function and cellular redox signalling in atherosclerosis.

The generation of a new mouse model for targeted *Gch1* deletion in atherosclerosis enables important new questions to be addressed on the requirement for BH4. Genetic or pharmacological increases in BH4 have shown that supra-physiological levels of BH4 can inhibit plaque progression. However, it is unknown whether BH4 deficiency alone is sufficient to alter plaque biology. Previous models of BH4 deficiency are either limited by very extreme global phenotypes^24^^,^^25^ or embryonic lethality^26^ which prevents the study of atherosclerosis. The Hph-1 mouse, which has variable systemic BH4 deficiency in different cell types,^27^^,^^28^ has not been assessed for atherosclerosis and hence to date the impact of loss of BH4 on plaque progression is unknown. We have now shown that cell-specific deficiency in endothelial cell and leucocyte BH4 is sufficient to accelerate atherosclerosis in ApoE^–/–^ mice. In order to evaluate the contribution of specific cell types in the progression of atherosclerosis, we generated bone marrow chimeras in which *Gch1* was selectively deleted in either endothelial or leucocytes. We found that loss of *Gch1* from either cell type alone was insufficient to accelerate atherosclerosis, indicating that *Gch1* from both endothelial cells and leucocytes was required for accelerated plaque formation. BH4 levels and nitrate production in BMDM from the chimeric mice confirmed correct bone marrow reconstitution. Although this demonstrates that all cells derived from the recipient bone marrow after chimerization were derived from the donor mice, we cannot exclude the possibility that the selection pressures of bone marrow reconstitution may impact plaque progression.

Loss of eNOS function and the resultant loss of NO in the eNOS^–/–^ mouse leads to endothelial cell dysfunction, increased macrophage recruitment via increased vascular VCAM-1 expression and ultimately accelerated atherosclerosis.^29^ In the current study we now show a similar pattern of endothelial cell dysfunction with an enhanced contractile function in chow fed mice driven by a decrease in NOS dependent relaxation and blunted endothelial cell mediated dilation in the aorta. HFD feeding resulted in an even more pronounced blunting in endothelial cell mediated dilation in *Gch1*^fl/fl^Tie2CreApoE^–/–^ mice which was fully reversible with *ex vivo* incubation with the BH4 precursor, sepiapterin, indicating that this defect was mediated by BH4 deficiency. Increased aortic VCAM-1 expression was also observed in both chow and HFD fed *Gch1*^fl/fl^Tie2CreApoE^–/–^ mice. This is in keeping with previous studies where we demonstrated that pharmacological supplementation of BH4 decreased aortic VCAM-1 expression.^27^ Furthermore, in HFD *Gch1*^fl/fl^Tie2CreApoE^–/–^ expression of the pro-atherogenic cytokine TNFα was significantly increased, indicating a more atherogenic environment. Interestingly, eNOS mRNA expression was also increased in HFD mice, possibly due to a compensatory upregulation mediated by H_2_O_2_.^30^^,^^31^ Increased blood pressure was observed in both male and female chow fed *Gch1*^fl/fl^Tie2CreApoE^–/–^. The endothelial cell dysfunction observed in these mice could in part contribute to increase in blood pressure. However, in HFD mice the increase in blood pressure was observed only in male not female *Gch1*^fl/fl^Tie2Cre ApoE^–/–^, whereas endothelial cell dysfunction was clearly apparent in female HFD *Gch1*^fl/fl^Tie2CreApoE^–/–^ mice making it unlikely that changes in endothelial function are solely responsible for the change in blood pressure. Blood pressure control is dependent not only on vascular function but also baroreceptor function, sensitivity of the renin–angiotensin–aldosterone system, central control and kidney function. It is possible that alteration in one of these alternative control systems has resulted in a compensatory normalization in blood pressure.

Further to observations in the eNOS^–/–^ ApoE^–/–^ mouse, where endothelial cell dysfunction is attributed to loss of eNOS and hence NO production,^32^ we now show that preservation of eNOS, but selective loss of NO generation due to BH4 deficiency is associated with increased endothelial cell superoxide and ROS generation. It is likely that both decreased NO and increased ROS generation, due to eNOS uncoupling, contributed to the increase in atherosclerosis, as shown by a previous study where endothelial overexpression of eNOS increased NO but also increased superoxide, by eNOS uncoupling, resulting in increased atherosclerosis.^5^ This study confirmed the pathological role of uncoupled eNOS in atherosclerosis, since treatment with BH4 or crossing with mice with endothelial cell targeted *Gch1* overexpression re-coupled eNOS, resulting in a reduction in atherosclerosis.^5^^,^^6^

An important new finding in male *Gch1*^fl/fl^Tie2CreApoE^–/–^ mice is an increased contribution of macrophages to atherosclerotic plaques. This increase could be due to an increase in either recruitment or retention of macrophages. Importantly, there was no difference in the number of circulating monocytes in male *Gch1*^fl/fl^Tie2CreApoE^–/–^ mice. The difference in macrophage content of the plaques could be due to an increase in the migratory capacity of *Gch1*-deficient macrophages. However, this is unlikely to be the mechanism, since we found no difference in *in vivo* macrophage chemotaxis between the two groups. The increase could also be due to increased retention of macrophages within the plaque. We observed decreased Cxcl5 expression in LPS/IFNγ stimulated macrophages from *Gch1*^fl/fl^Tie2CreApoE^–/–^ mice. Cxcl5 is important for macrophage turnover within the plaque, with a previous study showing that antibody-mediated inhibition of Cxcl5 increased macrophage retention in plaques.^33^ Interestingly, increased macrophage content was not observed in plaques from female *Gch1*^fl/fl^Tie2CreApoE^–/–^ mice. This difference could be due to the well-known difference in the time course of plaque progression observed between male and female ApoE^–/–^ mice. In addition to macrophages, mast cells have been demonstrated to have a role in plaque progression.^34^^,^^35^*Gch1* expression is regulated in mast cells and has been shown to have an important role in the release of NO and serotonin.^36–38^ However, the increase in atherosclerosis observed in *Gch1*^fl/fl^Tie2CreApoE^–/–^ mice is unlikely to be mediated by changes in mast cells, since no differences were observed in either total, granulated or de-granulated mast cell numbers.

The deletion of *Gch1* in macrophages in *Gch1*^fl/fl^Tie2CreApoE^–/–^ mice allows, for the first time, the selective investigation of the effects of iNOS regulation by BH4 *in vivo*. In contrast to eNOS, which has an atheroprotective role, iNOS has been shown to be pro-atherogenic; iNOS^–/–^ mice are protected from atherosclerosis, with a greater impact observed in more advanced lesions.^8–11^ In plaques iNOS is predominantly co-localized to macrophages, with the pro-inflammatory role of iNOS ascribed to the high-level output of NO, which reacts with ROS to form reactive nitrogen species. Strikingly, we observed that inflammatory BMDMs from *Gch1*^fl/fl^Tie2CreApoE^–/–^ mice had almost complete loss of NO production, as measured by nitrite production. However, this loss of NO production was accompanied by increased ROS production, revealing a key role of *Gch1* in the control of macrophage cellular redox state. This finding is in contrast to BMDM from iNOS^–/–^ mice which have reduced NO production and reduced ROS production^12^ and reduced 3-nitrotyrosine staining in plaques.^12^ These differences raise the possibility that BH4 deficiency in inflammatory macrophages results in a more pathological phenotype than that observed in macrophages from iNOS^–/–^ mice. Alterations in macrophage ROS production has been previously shown to alter foam cell formation,^39^ with inhibition of iNOS altering the ability of macrophages to form foam cells.^40^ We observed that *Gch1* deficient inflammatory LPS/IFNγ stimulated macrophages formed significantly larger foam cells than macrophages from their littermate controls. This increase was not due to alterations in cholesterol efflux from the cell as reverse cholesterol transport was similar between genotypes. In contrast to macrophages from iNOS knockout mice, macrophages from Gch1^fl/fl^Tie2CreApoE^–/–^ mice had decreased NO and increased ROS, implicating iNOS dependent ROS in foam cell development. We observed a significant reduction in anti-oxidant genes in the absence of *Gch1*, *HO-1*, *Nqo1* and *Prdx1* are known to be Nrf2 related genes. However, interestingly the reduction in anti-oxidant genes was not associated with a reduction in Nrf2 nuclear translocation. This could be due to difference in the time course of Nrf2 nuclear translocation vs. gene expression or due to modulation of alternative pathways. Bach1 binds to heme-responsive elements inhibiting the binding of Nrf2,^41^ with global deficiency in Bach 1 associated with reduced atherosclerosis.^42^ Alternatively, the changes in gene expression could be mediated by an alternative pathway such as repression of Ndy1 which has been shown to modulate the expression of Nqo1.^43^ This significant decrease in anti-oxidant defence signalling may compound the cellular redox state caused by iNOS uncoupling, by decreasing activation of cellular redox defence mechanisms.

In this study, deficiency in endothelial cell and leucocyte BH4 was not sufficient to impact on circulating BH4 levels in chow fed mice, demonstrating that under these conditions circulating BH4 levels are determined by BH4 production by cells other than endothelial cells and leucocytes. However, after high fat feeding a significant reduction in circulating BH4 and BH2 levels was observed in both male and female *Gch1* knockout mice, indicating that under these conditions endothelial cell and leucocyte BH4 make a significant contribution to circulating levels. The change in plasma BH4 was accompanied by a decrease in liver BH4 levels in Gch1^fl/fl^Tie2CreApoE^–/–^ mice, which in turn could be a contributing factor to the reduction in circuiting biopterin levels observed in this mouse. In both genotypes, HFD increased liver BH2 levels with a greater increase observed in liver from Gch1^fl/fl^Tie2CreApoE^–/–^ mice, suggesting increased oxidation of BH4 and/or decreased recycling of BH2 back to BH4, by DHFR. In addition to these changes in liver biopterins, aortic BH4 levels in HFD *Gch1*^fl/fl^ApoE^–/–^ were increased compared with chow fed animals, which may in part explain the preservation of circulating BH4 levels in WT mice. There was no difference in circulating BH4 levels in either male of female chimeric mice with loss of *Gch1* in either endothelial cells or leucocytes. This indicates that loss of *Gch1* in both endothelial cells and leucocytes is required for the reduction in circulating BH4 levels in HFD animals. It might be expected that plasma biopterin levels would increase after HFD as previous studies in humans have shown a positive correlation between plasma BH4 levels and systemic inflammation.^15^ It could be that systemic inflammation observed in ApoE^–/–^ mice^44^ is a sufficient stimulus to elevate plasma BH4 levels and that HFD is not additive to this effect.

In conclusion, we have shown for the first time a key role for endothelial cell and leucocyte *Gch1* and BH4 in the progression of atherosclerosis. Our study highlights the importance of both endothelial cell and macrophage BH4 in the regulation of NOS function and cellular redox signalling in atherosclerosis. These novel findings reveal contrasting endothelial cell and leucocyte requirements for *Gch1* and BH4 in the progression of atherosclerosis and identify new BH4-dependent determinants of inflammatory cell redox signalling and foam cell formation.

## Supplementary material

Supplementary material is available at *Cardiovascular Research* online.

**Conflict of interest:** none declared.

## Funding

British Heart Foundation Programme Grant (RG/12/5/29576), Chair award (CH/16/1/32013), and Oxford British Heart Foundation Centre of Research Excellence (RE/13/1/30181). Wellcome Trust (090532/Z/09/Z) and the National Institute for Health Research (NIHR) Oxford Biomedical Research Centre.

## Supplementary Material

Supplementary Data
