# Supplementary material for: Roles for endothelial cell and macrophage Gch1 and tetrahydrobiopterin in atherosclerosis progression
Source: Cardiovasc Res. 2018 Mar 27;114(10):1385–99. doi: 10.1093/cvr/cvy078 (PMC6054219; doi:10.1093/cvr/cvy078)
Supplement: Supplementary Data [file cvy078_supplementary_figure_legands.docx]

**Supplementary Figure 1**: **A**, BH2 levels in aortas of chow or HDF mice showing a significant increase in BH2 levels in *Gch1*^fl/fl^ApoE^-/-^ aortas from HFD mice (two way ANOVA, n=7-12 per group). **B**, Representative immunoblot showing expression of GTPCH in liver from *Gch1*^fl/fl^Tie2CreApoE^-/-^ mice. **C**, A significant reduction in liver BH4 levels was observed in *Gch1*^fl/fl^Tie2CreApoE^-/-^ mice after HFD (two way ANOVA, n=5-11 per group). **D,** A significant increase in BH2 levels was observed in the liver from HFD *Gch1*^fl/fl^ApoE^-/-^ and *Gch1*^fl/fl^Tie2CreApoE^-/-^ mice with a greater increase observed in livers from *Gch1*^fl/fl^Tie2CreApoE^-/-^ mice (two way ANOVA, n=5-6 per group). **E**, BH2 levels in primary endothelial cells, a significant reduction in BH2 levels was observed in endothelial cells from *Gch1*^fl/fl^Tie2CreApoE^-/--^ mice, (Mann Whitney U test n=4-6 per group). **F**, BH2 levels in unstimulated and LPS/IFNγ stimulated macrophages, a significant increase in BH2 levels was observed in stimulated BMDM from *Gch1*^fl/fl^ApoE^-/-^ compared with unstimulated macrophages. A significant reduction in BH2 levels was observed in stimulated BMDM from *Gch1*^fl/fl^Tie2CreApoE^-/-^ compared with stimulated macrophages from *Gch1*^fl/fl^ApoE^-/-^ mice. (two way ANOVA, n=8 per group). * = P<0.05 between genotypes of the same treatment, $ = P>0.05 between treatments of the same genotype. Black symbols=*Gch1*^fl/fl^ApoE^-/-^, red symbols=*Gch1*^fl/fl^Tie2CreApoE^-/-^.

**Supplementary Figure 2**: Heart rates in male and female mice fed a chow (**A**) or high fat diet (**B**). There was no significant difference in heart rates between genotypes (Un-paired T test, n=5-14). Black symbols=*Gch1*^fl/fl^ApoE^-/-^, red symbols=*Gch1*^fl/fl^Tie2CreApoE^-/-^.

**Supplementary Figure 3**:Mast cell quantification in aortic root. **A**, Representative images of mast cells in the perivascular/adventitia area of the aortic roots from male and female *Gch1*^fl/fl^Tie2Cre ApoE^-/-^ mice and their littermate controls. Total, granulated and degranulated mast cell numbers in male (**B**) and female (**C**) mice, high power images of individual mast cells are shown in black boxes. There was no significant difference in mast cell numbers between groups, Un-paired T test, n=12-18 per group. Black symbols=*Gch1*^fl/fl^ApoE^-/-^, red symbols=*Gch1*^fl/fl^Tie2CreApoE^-/-^.

**Supplementary Figure 4: Aortic vasoconstrictor responses. A,** Vasoconstriction to phenylephrine (PE) was increase in chow fed female *Gch1*^fl/fl^Tie2CreApoE^-/-^ mice. L-NAME (100 µmol) caused a significant increase in contractile response in both groups with no difference in genotype observed after incubation with L-NAME (RM ANOVA; n=7). **B**, No difference in vasoconstriction to phenylephrine (PE) was observed between genotypes in female HFD mice either before or after incubation with sepiapterin (RM ANOVA; n=8 per group). * = P<0.05 between genotypes of the same treatments. Black symbols=*Gch1*^fl/fl^ApoE^-/-^, red symbols=*Gch1*^fl/fl^Tie2CreApoE^-/-^.
